# The intestinal microbiota of lake anchovy varies according to sex, body size, and local habitat in Taihu Lake, China

**DOI:** 10.1002/mbo3.955

**Published:** 2019-11-29

**Authors:** Min Jiang, Mengyuan Xu, Congping Ying, Denghua Yin, Pei Dai, Yanping Yang, Kun Ye, Kai Liu

**Affiliations:** ^1^ Scientific Observing and Experimental Station of Fishery Resources and Environment in the Lower Reaches of the Changjiang River Ministry of Agriculture and Rural Affairs Freshwater Fisheries Research Center CAFS WuXi China; ^2^ Wuxi Fishery College Nanjing Agricultural University Wuxi China

**Keywords:** fish body size, intestinal microbiome, lake anchovy, local habitat, sex, Taihu Lake

## Abstract

Lake anchovy (*Coilia ectenes taihuensis*) is a sedentary, dominant fish species that forms an unmanaged fishery in Taihu Lake, eastern China. The environment and developmental stage of lake anchovy are likely important drivers of their gut microbiome, which is linked to host health and development*.* To investigate the relationship between the gut microbiome and three defined factors (fish sex, fish body size, and the local habitat), high‐throughput sequencing of the 16S ribosomal RNA gene was used to study the microorganisms of 184 fish samples and four water samples collected in Taihu Lake. Four dominant bacterial phyla (Proteobacteria, Firmicutes, Planctomycetes, and Cyanobacteria) were present in all fish samples. We compared the microbial communities of males and females and found that the relative abundance of *Corynebacteriaceae* was significantly higher in males than in females, while the opposite trend was detected for *Sphingomonadaceae*. We also discovered that the relative abundance of Firmicutes was positively correlated with fish body size and that the proportions of Proteobacteria and Tenericutes were lower in larger fish than in fish of other sizes. Finally, we found that the difference in microbial richness between eastern and northern Taihu Lake was the most marked. Lake anchovy was rich in *Lactobacillus* and *Clostridium* in the eastern site, while those in the northern site had the highest abundance of *Sphingomonas* and *Methylobacterium*, suggesting that the local habitat may also influence the intestinal microbiome. These findings will not only help researchers understand the community composition of the intestinal microflora of lake anchovy but also contribute to the protection of fish resources in Lake Taihu and the sustainable use of lake anchovy.

## Graphical abstract

The present study documented the basic composition of the gut microbiota in lake anchovy (*Coilia ectenes taihuensis*) in Taihu Lake, China, and investigated the potential factors that influence variation in microbial community composition. Three potential factors may affect the intestinal microbiota of lake anchovy: sex, body size, and local habitat.

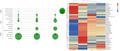

## 1 INTRODUCTION

In recent years, the study of the intestinal microbial community composition and structure and the interactions of microbial communities with their vertebrate hosts have developed rapidly. These microorganisms play crucial roles in the metabolism, the immune response, and the normal development of their hosts (Joyce & Gahan, 2014; Kobyliak, Virchenko, & Falalyeyeva, 2015; Nicholson et al., 2012). Within fish, the composition of the intestinal microflora varies greatly depending on the genetic background (Ley et al., 2008), diet (Bolnick et al., 2014; Dawood, Koshio, Ishikawa, & Yokoyama, 2015; Michl et al., 2017), body size (Briones et al., 2012; Davis, Blanchette, Pusey, Jardine, & Pearson, 2012; Ringo & Birkbeck, 1999), and environment of the fish (Lv, et al., 2018; Roeselers et al., 2011; Yoshimizu, Kimura, & Sakai, 1976). Understanding the effects of these variables on intestinal microbial communities is critical for fundamental biology and the management of fisheries.

The environment and genetics both affect the gut microbiome. Habitats may vary in local prey abundance and availability (due to eutrophication and fish body size), which are both important factors determining fish foraging habits (Sha, Su, Zhang, Zhang, & Xu, 2015), and lake anchovy exhibit diverse dietary strategies at both the population and individual levels depending on their environment (Chen & Zhu, 2008). Diet, thus, serves as a source of both bacteria and variation in the nutritional environment of the intestines. In terms of genetics, host sex affects the intestinal microbial composition largely by way of sex hormones (Markle et al., 2013). Studies in mammals have shown that sex hormones can modulate microbiota compositions (Omry et al., 2012).

Taihu Lake is the third‐largest freshwater lake in China and is situated in the Changjiang (Yangtze) Delta, the most industrialized area in China with a high population density, high urbanization, and high economic development. Taihu Lake is important for water supply, flood control, tourism and recreation, shipping, and aquaculture (Qin, Xu, Wu, Luo, & Zhang, 2007). Environmental indicators of Taihu Lake water, such as the transparency, permanganate index, nitrogen and phosphorus concentrations, and chlorophyll concentration, tend to show significant spatiotemporal variation (Zhu, 2009). For example, chlorophyll‐a is characteristic of cyanobacterial blooms and is mainly concentrated in the north and northwest areas of the lake (Liu, Yang, Gao, & Jiang, 2011).

Lake anchovy (*Coilia ectenes taihuensis*, Engraulidae, Clupeiformes), is a small‐to‐medium size omnivorous fish. It is the dominant fish in Taihu Lake (Liu, Bao, Wu, & Cao, 2007). Lake anchovy does not migrate and live their entire life in the lake (Sciences, 1990). Their feeding habit depends on body size, season (Yu, He, Li, Chen, & Liu, 2012), and local habitat (Duan, Xu, Liu, Zhou, & Xu, 2017). Lake anchovy feeds mainly on zooplankton, juvenile shrimp, juvenile fish, and aquatic insects, with increasingly larger prey as they grow in size. Smaller fish (<130 mm) feed primarily on cladocerans and copepods, whereas larger fish (131–170 mm) also feed on fish and shrimp (up to 19% of their prey). The largest fish (>170 mm) feed primarily on fish and shrimp (50%–100% of prey; Tang, 1987). Fish often develop specific foraging strategies according to different local conditions (Briones et al., 2012). For example, habitat nutrition can indirectly affect the dietary composition of lake anchovies through complex prey responses, which limits the coupling of food webs and affects energy flux and nutrition dynamics (Sha et al., 2015).

To date, studies of *Coilia* species have focused on catch, morphological differences, growth, development, and reproduction (Jiang, Yang, Liu, & Shen, 2011; Liu, Bao, & Wan, 2008; Tang, 1986). Relatively few studies have investigated the intestinal microbiota of lake anchovy (Nie, Xu, Cheng, et al., 2015; Nie et al., 2014; Nie, Xu, Du, et al., 2015). Moreover, little is known about the factors that drive variation in their intestinal microbiome. Therefore, the objectives of our study were (a) to investigate the community structure and species composition of the intestinal microbiota of lake anchovy; (b) to explore the factors that influence community diversity and structure of the intestinal microbiome; and (c) to compare differences in intestinal microbiome in fish of different sexes, body sizes, and local habitats*.*

## 2 MATERIALS AND METHODS

### 2.1 Sampling

**Figure 1:**
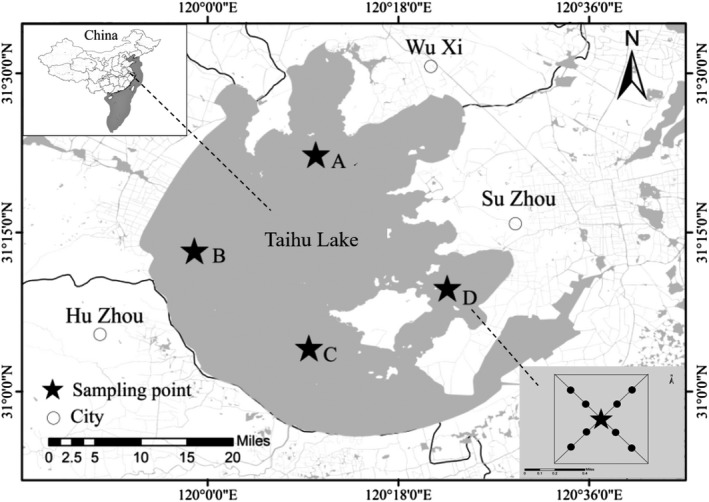
The four sampling sites in Taihu Lake. Fish were sampled from the sites in the north (a, 31°19′18″N–31°22′33″N, 120°10′9″E–120°13′16″E, *n* = 48), west (b, 31°12′50″N–31°15′20″N, 119°59′57″E–120°2′26″E, *n* = 44), south (c, 31°3′31″N–31°6′11″N, 120°6′55″E–120°10′9″E, *n* = 45), and east (d, 31°9′17″N–31°11′18″N, 120°21′49″E–120°24′34″E, *n* = 47) of the lake

We collected 184 lake anchovy individuals from sampling stations established during the major fishing season (September) in 2017 from four different regions of Taihu Lake (Figure 1). Each sampling station had six gill nets with different mesh sizes. The total net length was 750 m, and the net height was 1.5 m. The mesh sizes of the gill nets were 1.2, 2, and 4 cm (each 15‐m long), and 6, 8, 10, and 14 cm (each 20‐m long), for a total net length of 125‐m long.

**Figure 2:**
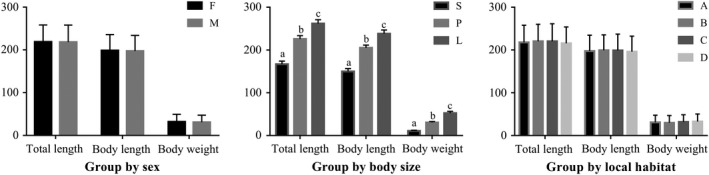
Morphological measurement data statistics for all fish samples. Fish were grouped by sex (F = female, M = male); body size (S = small, about 10 g, P = medium, about 30 g, and L = large, about 50 g); and lake region (A = northern part of Taihu Lake, B = western, C = southern, D = eastern). Letters in each panel indicate significant differences between groups (ANOVA, *p* < .001)

The collected fish were weighed and sexed (male or female) and assigned to one of three groups based on body size, as determined from preliminary data: S (c. 10 g), P (c. 30 g), or L (c. 50 g) **(**Figure 2). The whole intestinal tract of each fish was dissected on ice using sterile scissors, placed in an aseptic cryopreservation tube, rinsed with sterile water and, after the perienteric fat was removed, instantly frozen with liquid nitrogen. A total of 44–48 fish gut samples were collected from each part of Taihu Lake (Figure 1). Biological measurements of all samples are included in the Supplementary Materials (Table A1).

Water samples were also collected from each sampling site. We took 10 water samples from each of the four sampling sites. Two samples were collected at the center of the sampling site, and eight were collected along the diagonals of the 1,000 m × 1,000 m region centered on the site (Figure 1, see subpanel near D). At each sampling site, we used a 0.5‐L water collector at a depth of 0.5 m. Each sample was fully mixed and then packed into a 50‐ml aseptic centrifuge tube. Samples were then filtered onto polyester cellulose filters (Millipore) with a pore size of 0.2 μm. Filters from the same sampling site were stored in the same centrifuge tube filled with anhydrous ethanol and immediately frozen at −80°C until DNA extraction.

### 2.2 DNA extraction, 16S rRNA gene amplicon preparation, and sequencing

The water samples were processed to extract DNA as follows. The filter was cut into pieces in a sterile 50‐ml centrifuge tube using a sterile scalpel. DNA extraction was performed with the E.Z.N.A. Water DNA Kit (OMEGA Bio‐Tek) according to the manufacturer's protocol. The whole intestine of each fish was cut into pieces with sterile scissors and tweezers and placed into a 2‐ml centrifuge tube on ice. DNA extraction was performed with the E.Z.N.A. Stool DNA Kit (OMEGA Bio‐Tek).

DNA integrity was verified using 0.8% agarose gel electrophoresis, and the quantitative analysis of DNA was carried out by ultraviolet spectrophotometry. The hypervariable V4‐V5 regions of the 16S rRNA gene, which are frequently targeted for evaluating bacterial communities (Martínez‐Porchas, Villalpando‐Canchola, & Vargas‐Albores, 2016), were directly amplified from 10 ng of total DNA with PAGE‐purified Illumina platform‐compatible adaptor oligos that contained features such as sequencing primers, sample‐specific barcodes, and 16S PCR primers (forward primer, 515F: GTGCCAGCMGCCGCGGTAA (Caporaso et al., 2011); reverse primer, 907R: CCGTCAATTCMTTTRAGTTT (Lozupone et al., 2013)). PCR was performed using the following cycling profile: initial denaturing at 95°C for two min followed by 20 cycles of 95°C for 30 s, 60°C for 30 s, and 72°C for 6 s. The PCR amplification products were detected by 2% agarose gel electrophoresis, and the target fragments were reclaimed with a kit. The Gel Recovery Kit (Axygen) was used for recovery. We quantified the PCR‐amplified and ‐recovered products with the fluorescence reagent Quant‐iT PicoGreen dsDNA Assay Kit. The DNA concentration was then quantified using a microplate reader (BioTek, FLx800). The purified amplicons from individual samples were pooled in equal mass (molar) ratios. We prepared a sequencing library from the TruSeq Nano DNA LT Library Prep Kit (Illumina Company). First, the library was examined by Agilent Bioanalyzer using the Agilent High Sensitivity DNA Kit. Then, the library was quantified with the fluorescence quantitative system using the Quant‐iT PicoGreen dsDNA Assay Kit. The library pool was sequenced using an Illumina MiSeq Reagent Kit V3 on an Illumina MiSeq sequencer.

### 2.3 Bioinformatics and data analysis

The raw data were sorted into independent files according to unique tags. After the removal of tags and primers, pair‐ended sequences were merged (Caporaso et al., 2010). Quality filtering included three steps. We discarded sequences (a) that contained “N”, (b) with a quality score less than 20 (finger base average sequencing accuracy ≥99%), and (c) with a length shorter than 150 bp. All sequences were classified into OTUs at a threshold of 97% similarity using the UPARSE pipeline (Edgar, 2013), and representative sequences of each OTU were generated simultaneously.

QIIME software (v1.8.0) (Caporaso et al., 2010) with UCLUST (Edgar, 2010) was used to merge the sequences obtained above and to partition OTUs according to a sequence similarity of 97%. The sequence with the highest abundance in each OTU was selected as the representative sequence of the OTU. The taxonomic information corresponding to each OTU was obtained by comparison with the Greengenes database (Release 13.8; Desantis et al., 2006) and by comparing the representative OTU sequence with the template sequence of the corresponding database from QIIME (v1.8.0).

All statistical analyses were conducted using R software v3.2.5 (R development Core Team, 2015). Alpha diversity indices, including Chao (1984), Simpson (1972), and Shannon (1948), the ACE estimator (Dawid, 1993) were calculated within QIIME for all samples. ANOSIM was used to verify the effectiveness of the grouping, and PERMONOVA was used to test whether the dissimilarity of microbial abundance among the different groups was significant. The OTU relative abundance values were analyzed using the linear discriminant analysis (LDA) effect size (LEfSe) algorithm (Segata et al., 2011).

To predict the potential functions of each sample based on 16S rRNA sequencing data, we used PICRUSt (Langille et al., 2013). We used the KEGG database and performed closed reference OTU picking, using the sampled reads against a Greengenes reference taxonomy. The 16S copy number was then normalized, molecular functions were predicted, and the final data were summarized into KEGG pathways. The Kruskal–Wallis test was performed to determine whether the differences in metabolic function between groups were statistically significant. The differences in the predicted molecular functions of the bacterial communities among different groups were shown in a histogram using the “vegan” package (Ramette, 2007) in R.

## 3 RESULTS

### 3.1 Overview of the gut microbial community structure in lake anchovy

**Figure A1:**
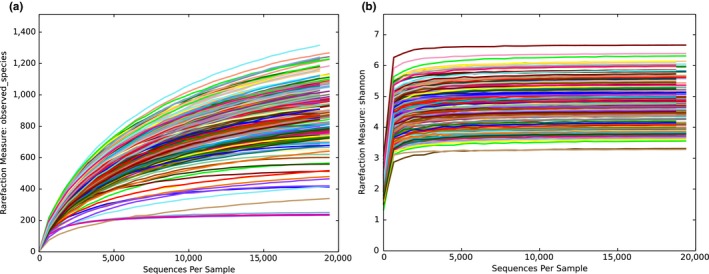
Rarefaction curves of all samples. The two panels correspond to observed‐species (a) and Shannon index rarefaction curves (b) of all samples

**Figure A2:**
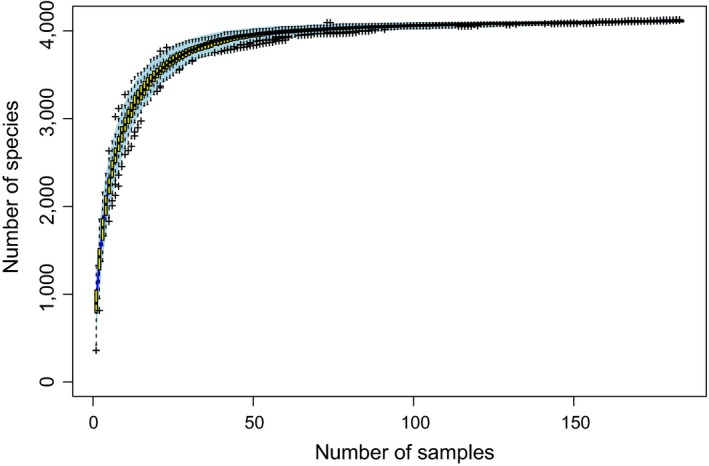
Species accumulation curve

Following quality trimming and chimera detection, a total of 6,842,704 valid reads and 4,208 OTUs were obtained from the 188 samples (Table A2), with an average of 35,687 reads per barcoded sample. The observed species and Shannon index rarefaction curves plateaued in all samples (Figure A1), indicating that most of the microbial diversity in these samples was captured by this sequencing analysis. The species accumulation curves also suggested that the sampling quantity was sufficient for analysis (Figure A2). Thus, it is unlikely that any undetected rare species would affect our conclusions.

**Figure 3:**
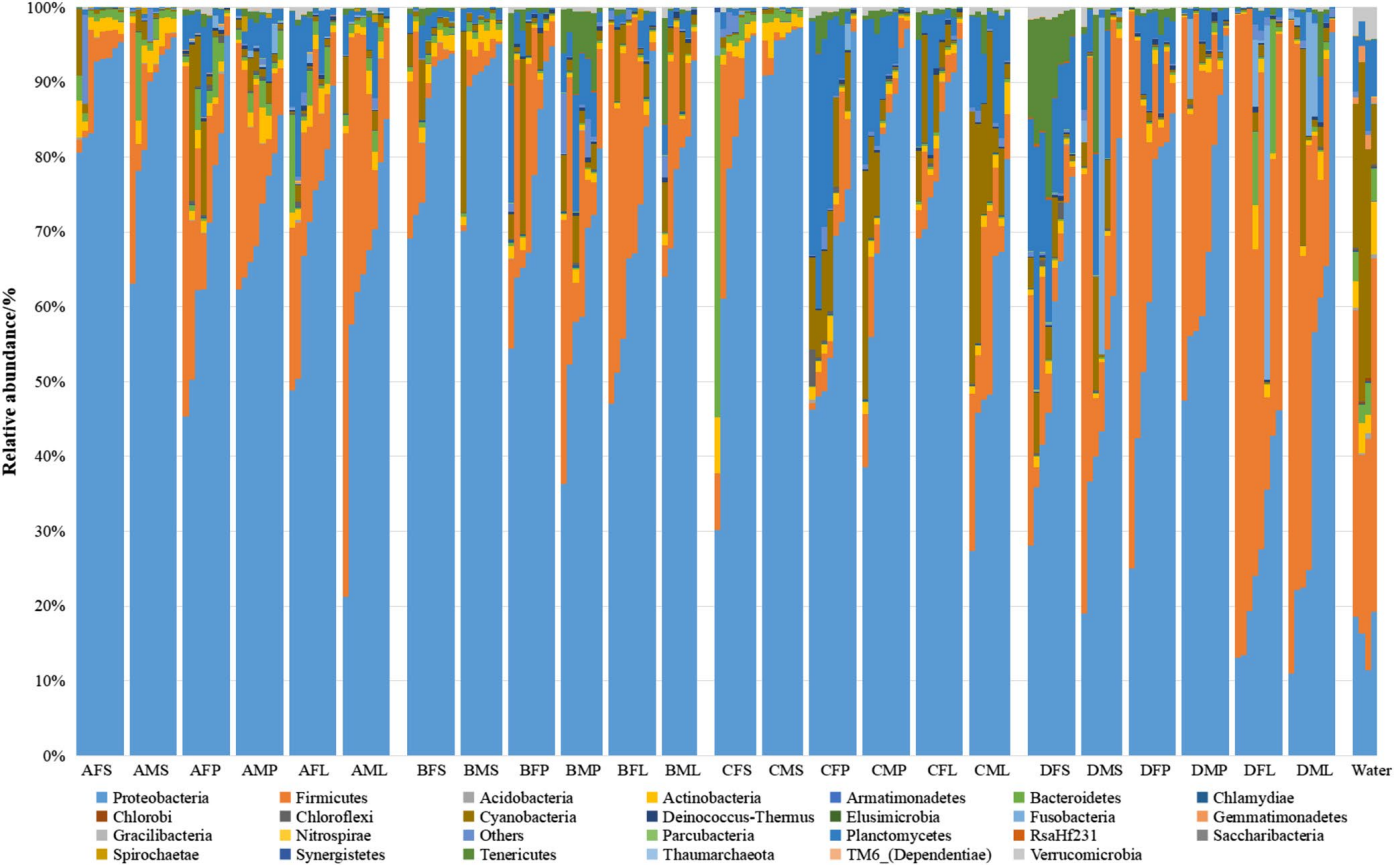
Phylum‐level gut microbiota composition of lake anchovy. Three‐letter codes for each column indicate sample point, fish sex and fish body size. Fish were grouped by sex (F = female, M = male); body size (S = small, about 10 g, P = medium, about 30 g, and L = large, about 50 g); and lake region (A = northern part of Taihu Lake, B = western, C = southern, D = eastern). For example, AFS = fish from site A, of female sex, and small size. The water column (farthest right) shows samples from each site a, b, c, and d

**Figure A3:**
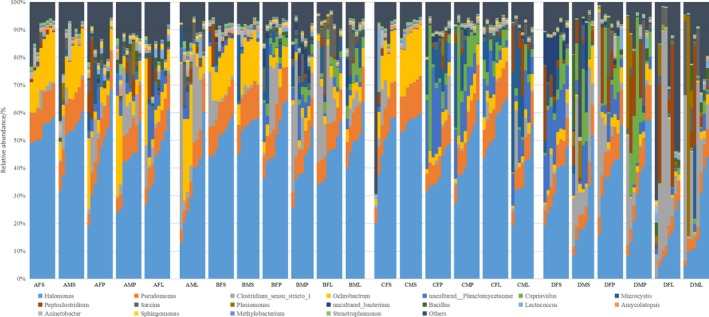
Dominant genera composition of lake anchovy. Three‐letter codes for each column indicate sample point, fish sex and fish body size. Fish were grouped by sex (F = female, M = male); body size (S = small, about 10 g, P = medium, about 30 g, and L = large, about 50 g); and lake region (A = northern part of Taihu Lake, B = western, C = southern, D = eastern). For example, AFS = fish from site A, of female sex, and small size. The water column (farthest right) shows samples from each site A, B, C, and D

A total of 490 genera belonging to 26 phyla were identified in the gut samples. The dominant phyla in the intestinal samples were Proteobacteria (69.0% relative abundance) and Firmicutes (16.4%), followed by Planctomycetes (4.8%), Cyanobacteria (3.4%), Tenericutes (1.5%), Actinobacteria (1.4%), and Bacteroidetes (1.3%). A total of 62 classes were identified. Proteobacteria largely consisted of Gammaproteobacteria (54.3%), Alphaproteobacteria (9.5%), and Betaproteobacteria (5.1%). Firmicutes were mainly composed of *Clostridia* (13.6%) and *Bacilli* (2.6%). The dominant genera were *Halomonas* (41.4%), *Pseudomonas* (9.3%), *Clostridium *sensu* stricto 1* (8.1%), *Ochrobactrum* (7.5%), *Uncultured Planctomycetaceae* (4.4%), *Cupriavidus* (3.4%), and *Microcystis* (3.0%; Figure A3). The relative abundance of major bacterial phyla in the intestinal content among the four sites was uneven (Figure 3).

In the water samples, a total of 34 phyla were detected (Figure 3, water). Firmicutes were the dominant bacterial phylum in all regions, followed by Cyanobacteria and Proteobacteria. The composition and richness of water microorganisms in the four regions of the Taihu Lake were different. For example, Firmicutes was most abundant in region D (50.4%) and the least abundant in region B (25.0%), while that of Planctomycetes was most abundant in region C (12.0%) and least abundant in region B (2.8%). Cyanobacteria were relatively less abundant in region D, whereas Proteobacteria were relatively more abundant. The microbiota composition of regions B and C showed more similarity than other region pairs.

### 3.2 Intestinal microbiota of female versus male lake anchovy

**Figure 4:**
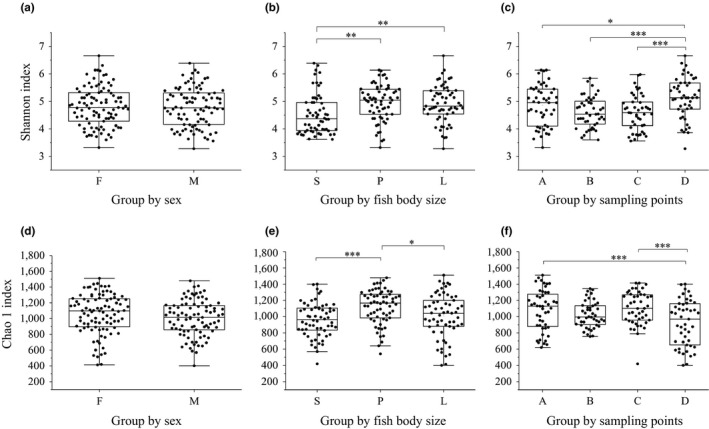
Variation in α‐diversity of gut microbiota of lake anchovy. The Shannon diversity index (Figure 2a,b, (c) and Chao1 richness estimator (Figure 2d,e,f) are presented. Fish were grouped by sex (a, d; F = female, M = male); body size (b,e; S = small, about 10 g, P = medium, about 30 g, and L = large, about 50 g); and lake region (c,f; A = northern part of Taihu Lake, B = western, C = southern, D = eastern). Comparisons among groups were made by a one‐way ANOVA. Statistically significant differences are indicated with asterisks: *p* < .05(*), *p* < .01(**), *p* < .001(***)

Female and male fish had the same dominant bacteria, and there was no significant difference in the relative abundance of the four most dominant phyla between the sexes (*p* > .05 for all). There is no difference between the two groups of samples based on ANOSIM (*R* = 0.049, *p* = .203). Alpha diversity indices (Shannon index and Chao1 index) confirmed that there was no significant difference in gut microbial diversity or richness between males and females (ANOVA, Shannon index: *p* = .78; Chao1 index: *p* = .124, Figure 4a,d).

**Figure 5:**
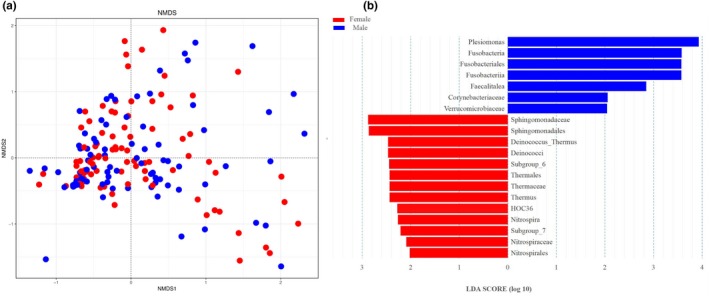
Variation in gut microbiota between male and female lake anchovy fish from Taihu Lake, China. (a) Partial least squares discriminant analysis showing differences in gut microbiota between female and male fish. (b) Twenty significantly discriminative taxa with absolute LDA score ≥2.0. F = female, M = male

NMDS revealed that some samples from male and female fish were no significantly different (Figure 5a). The difference in the microbial composition (relative abundance) of the gut microbiome may derive from the contrasting abundance of each taxon in the two groups, compared using the LEfSe algorithm. At a stringent cutoff value, the taxa displayed a significant difference in their abundance between males and females (absolute LDA score log_10_ ≥ 2.0, Figure 5b). For instance, both Fusobacteria and Deinococcus–Thermus were significantly different (*p *<* *.05). In addition, intersex differences were also identified at lower taxonomic levels. For example, at the family level, the relative abundance of Corynebacteriaceae was significantly higher in males than in females, while the opposite was observed for Sphingomonadaceae. At the genus level, significantly more reads were assigned to *Nitrospira* in female samples than in male samples.

### 3.3 Comparison of the gut microbiota across fish body sizes

There were significant differences among the three groups of body size based on the ANOSIM (*R* = 0.2732, *p* = .001). The diversity of the gut bacterial communities in the small lake anchovy was significantly higher than that of the large fish, as confirmed by Chao1 index (*p* < .05) and Shannon index (*p* < .005) analyses (Figure 4b,e). A clear distinction in the gut bacterial community structure of the different groups was also revealed by PERMANOVA analysis (*p* = .001).

**Figure 6:**
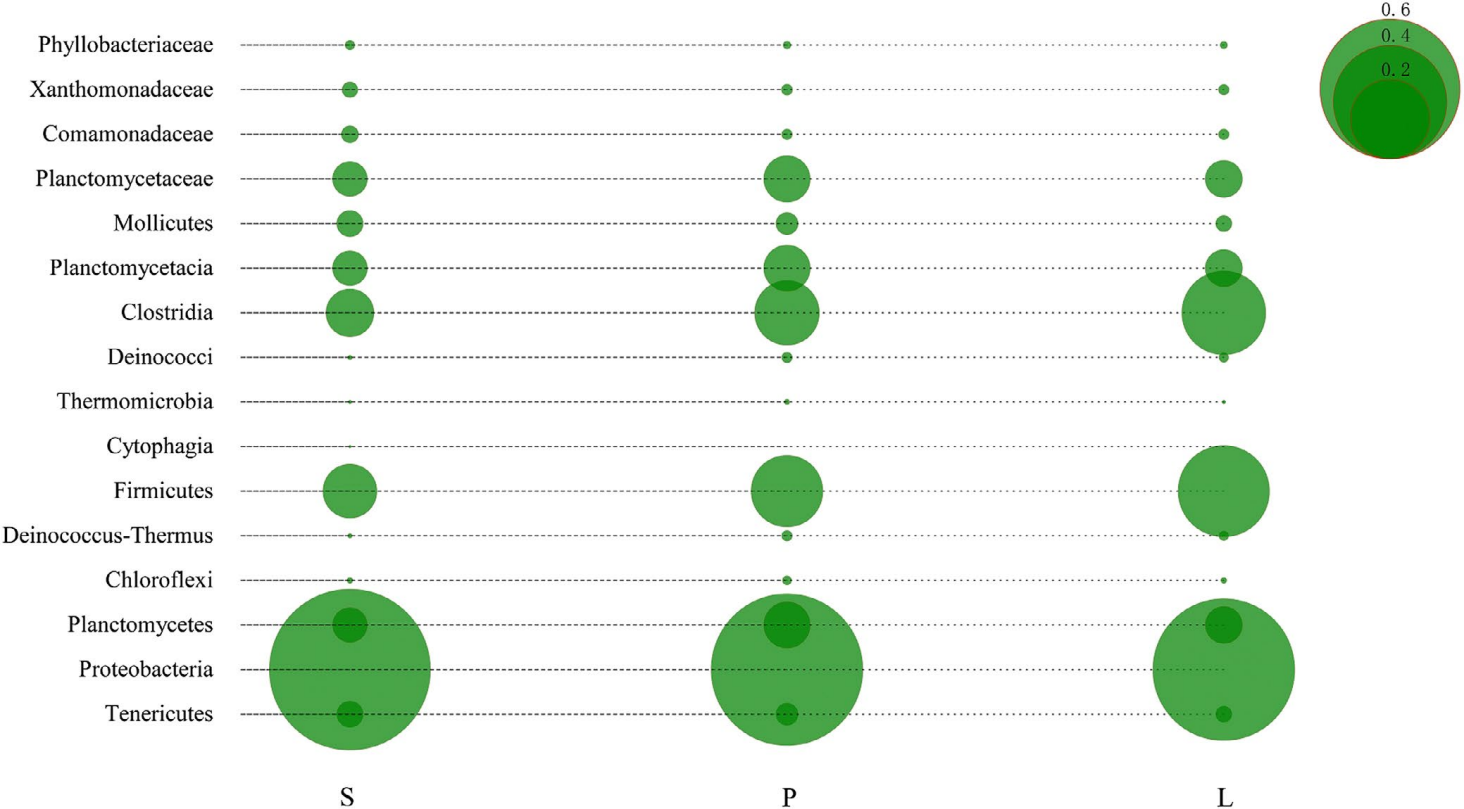
Selected microbial taxa displaying significant differences in relative abundance in the gut microbiome across fish of different body size. S = small fish, about 10 g, P = medium fish, about 30 g, and L =large fish, about 50 g. Relative abundance is indicated by the size of each circle

The relative abundance of the bacterial flora varied according to body size as shown by LDA. Proteobacteria, especially Comamonadaceae, were more abundant in small fish (0.9%) than in larger fish (0.3%; Figure 6). In contrast, the relative abundance of Firmicutes (8.7%–25.1%) was positively correlated with fish body size (Figure 6). The intestinal microbial communities of larger fish had a lower relative abundance of Tenericutes (0.8%) and higher relative abundance of Verrucomicrobia (0.3%) and Cyanobacteria (4.1%) than those of small fish, whereas Synergistetes were only detected in small fish.

### 3.4 Influence of geographic locations on the gut microbiota of lake anchovy

There were significant differences in microbial diversity among the different sampling sites (*p* < .05, ANOVA, Figure 4c,f). ANOSIM similarity analysis showed that there were significant differences among the four groups (*R* = 0.2285, *p* = .001). The gut microbiota was significantly more diverse in samples from site D than other sites (PERMANOVA, *p* = .001). Alpha diversity did not differ among samples from the A, B, and C sites.

**Figure 7:**
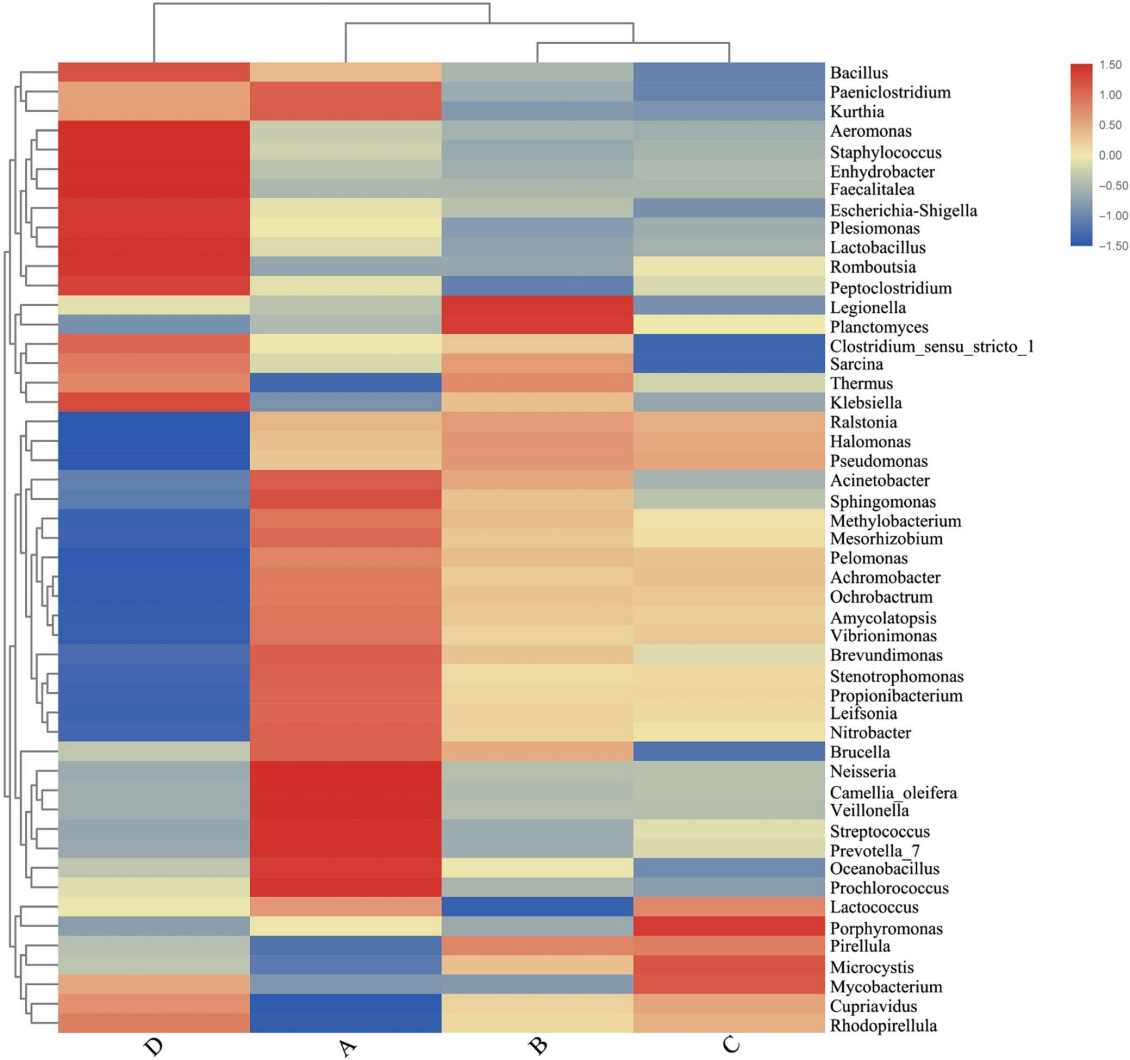
Heatmap analysis of the intestinal bacterial species sampled from four sites of Taihu Lake, China in September 2017. The color of the bar represents the abundance of each bacteria species in all samples. The longitudinal clustering indicates the similarity of all species among different groups. A = northern part of Taihu Lake, B = western, C = southern =, D = eastern

The differences in α‐diversity across the four sites may derive from the contrasting abundance of each taxon (Figure 7). The dominant genera in each site were different except for *Halomonas* and *Pseudomonas*. For example, *Stenotrophomonas* was more abundant in site A, and *Microcystis* was more abundant in site C than other sites. Site D samples had the lowest abundance of *Halomonas* (27.0%) and *Ochrobactrum* (2.4%), while *Clostridium_sensu_stricto_1* (15.6%) and *Cupriavidus* (6.4%) were more abundant in other regions than in site D. The microbial structure and richness of sites B and C were the most similar (Figure 7). Site B samples were lower in *Clostridium_sensu_stricto_1* (2.0%) and richer in *Planctomyces* (0.2%) than the other sites. *Lactobacillus* and *Bacillus* were particularly abundant in the gut microbiota of site D samples.

### 3.5 Biological pathways and functional categories inferred from the 16S data

**Figure 8:**
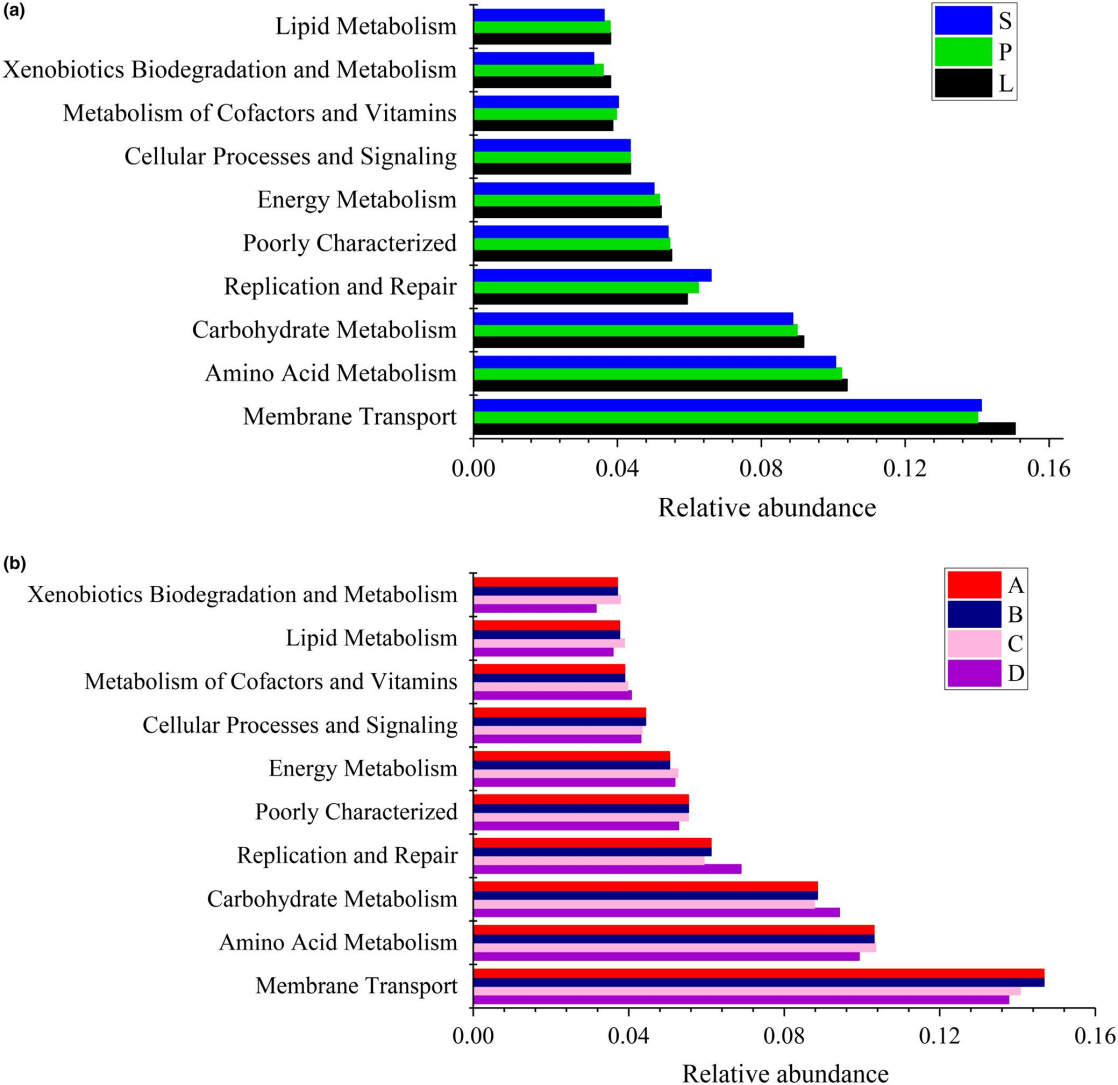
Functional predictions of different body size lake anchovy using PICRUSt. Histogram showing the ten dominant gene families across three body sizes (a) and four samplings sites (b) (level 2 KEGG Orthology groups). Size: S = small fish, about 10 g, P = medium fish, about 30 g, and L = large fish, about 50 g. Site: A = northern part of Taihu Lake, B = western, C = southern, D = eastern

**Figure A4:**
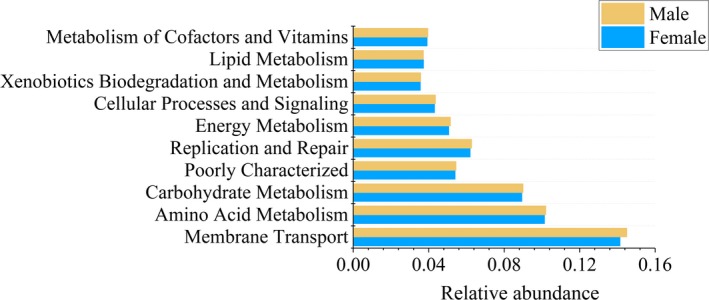
Functional predictions between sexes lake anchovy using PICRUSt. Fish were grouped by sex (F = female, M = male)

We used the PICRUSt algorithm to predict microbial functions that might be associated with the changes in OTU abundances detected via 16S sequencing. After adjusting for copy number variation in the 16S rRNA genes, a total of 6,909 KEGG (level 4 KOs) gene families (mean ± *SD* =5,526.3 ± 184.5 per sample) were identified from the OTU table. We identified 41 KEGG pathways, which exhibited similar gene functions but with some differences in abundance among the four different sampling sites and fish body sizes instead of sex (Figure A4). These included pathways related to membrane transport, amino acid metabolism, carbohydrate metabolism, replication and repair, energy metabolism, cellular processes and signaling, xenobiotic biodegradation and metabolism, and lipid metabolism, or poorly characterized pathways. The richness of xenobiotic biodegradation and metabolism, energy metabolism, and carbohydrate metabolism richness was greater in larger fish, while the richness of replication and repair richness was lower in larger fish than in smaller fish (Figure 8a). Among the ten dominant gene families noted above, the abundance of genes related to carbohydrate metabolism and nucleotide metabolism were higher in site D than other sites, while amino acid metabolism was lower in all other sites than in site D (Figure 8b).

## 4 DISCUSSION

In contrast to the extensive studies of the abundance and distribution of lake anchovy, little attention has been given to the distinctive gut microbiota of these fish. In our study, we explored the community composition and structure of the gut microbiota of lake anchovy.

### 4.1 Intestinal microbiota of lake anchovy

Our results characterized the molecular profile of microbial communities using high‐throughput sequencing technology. Proteobacteria was found to be the most dominant phylum across the intestine samples, similar to data from rainbow trout (Etyemez & BalcÃ Zar, 2015). Most notably, the high abundance of Proteobacteria in our results was largely due to a high number of Gammaproteobacteria, particularly *Halomonas*. *Halomonas* is one of the most predominant cultivated protease‐producing bacteria because of its chemoorganotrophic nature (Zhou et al., 2009), and omnivorous and carnivorous fish species are often enriched with this bacterium (Liu et al., 2016)*.* Previous studies found that Proteobacteria, Firmicutes, and Actinobacteria were predominant in grass carp (Wu et al., 2012). Furthermore, the guts of largemouth bass and bluegill were dominated by Fusobacteria, followed by Proteobacteria (Larsen, Mohammed, & Arias, 2014). In our study, the gut microbiota in lake anchovy was dominated by Proteobacteria, Planctomycetes, and Firmicutes and exhibited unique community structure and host‐specific compositions. Our results are consistent with previous results obtained using the denaturing gradient gel electrophoresis method (Nie, Xu, Du, et al., 2015). In addition, we also detected Cyanobacteria, Actinobacteria, Bacteroidetes, Chlamydiae, and other major bacteria.

### 4.2 Factors that affect the gut microbiome

Three potential factors may affect the intestinal microbiota of lake anchovy: fish sex, fish body size, and local habitat. Considering Taihu Lake in its entirety, we collected all samplings during the late breeding stage (Sun, 1987) of the same year; therefore, we temporarily excluded other factors and thus only considered the role of fish sex.

At the phylum level, we found that Fusobacteria was significantly more abundant in male fish than in female fish. Fusobacteria may be significantly promoted when animal proteins are included in the diet (Michl et al., 2017); because male lake anchovy undergoes more gonad development than females, they need to consume many high‐protein fish and shrimp. This dietary difference may explain the difference in Fusobacteria abundance between sexes.

There were several other significant differences in the gut microbial composition between sexes that may be related to immune function and/or sex hormones (Figure 5b). For example, male fish had a greater abundance of *Sphingomonas*. *Sphingomonas* has been reported to degrade microcystins (Hajime, Kiyomi, & Ken‐Ichi, 2009), which may be related to the higher immune function in males (Markle & Fish,^2014^). Host sex hormones and sex differences in immune function may also affect intestinal microbes (Markle & Fish, 2014; Omry et al., 2012), but based on our data, we cannot determine which of these mechanisms impacted the gut microbes. Nevertheless, our findings highlighted the importance of fish sex in structuring gut microbiomes.

Body size also influenced the microbiome composition of lake anchovy intestines*.* Gut microbiome diversity was lower in larger fish than in fish of other sizes, similar to *African turquoise killifish* (Smith et al., 2017). In large fish, the Firmicutes taxa were mostly composed of Clostridia at the class level and Lachnospiraceae at the family level. All known taxa in this family are strictly anaerobic and reside mainly within the digestive tracts of mammals (Kittelmann et al., 2013; María José et al., 2011; Meehan & Beiko, 2014). Some studies have shown that some species of this family can promote the digestion and utilization of proteins (Zhou, 2016). Larger fish have greater energy needs and feed on high‐protein foods, such as small fish and shrimp, to obtain sufficient energy. Thus, the enrichment of Firmicutes in the large fish group may contribute to energy intake and nutrient absorption. An increase in the abundance of Firmicutes has been observed during development from juveniles to adults (Hu et al., 2017).

At the genus level, the *Lachnospiraceae_NK4A136_group* content was highest in large fish, which is similar to Huang's findings (Wang et al., 2018). In small fish, significant differences in the distribution of Proteobacteria were also discovered, especially in the enrichment of Gammaproteobacteria (59.7%) and Alphaproteobacteria (13.8%). The high abundance of these two classes was due largely to the abundance of taxa in the families Xanthomonadaceae and Phyllobacteriaceae. Xanthomonadaceae are considered essential for the digestion of the host's nutritionally poor diet (Berg et al., 2016). Some species of these families may also exhibit cellulase activity and degrade a variety of aromatic compounds (Reid, Addison, Macdonald, & Lloydjones, 2011). The Phyllobacteriaceae family consists of environmental (soil and water) and plant‐associated bacteria, and some members of this family also exhibit cellulose‐degrading enzyme activity (Hameed et al., 2015). We hypothesize that small lake anchovy may eat *Microcystis* and other plants by mistake, and the enrichment of these bacterial families may facilitate cellulose degradation and nutrient absorption for smaller fish. However, although Cyanobacteria have been found in the stomachs of lake anchovy (Wang et al., 2016), there is no direct evidence for cellulose digestion in this species, and thus, our hypothesis requires further confirmation.

Similarly, the content of Comamonadaceae in small fish was significantly higher than that in other groups, similar to the findings of Huang, Li, Wang, and Shao (2016). In addition, we found strong similarity in the intestinal tract structure and richness between medium‐ and large‐sized fish. Although medium and large fish primarily consume fish and shrimp, medium‐sized fish also eat an abundance of other foods. Thus, different developmental stages of lake anchovy have specific dietary requirements, which lead to different microbial gut communities.

Overall, environmental factors may have the greatest impact on the lake anchovy gut microbiome. Comparison of the water and intestinal microbial community structures and relative abundances showed similarities, suggesting that the microflora in the water environment affect the gut microflora to some extent. Strikingly, some microbial abundances shared a one‐to‐one mapping between the intestinal tract and water environment. For example, Cyanobacteria was the least abundant both in water and intestine samples from site D. Conversely, the relative abundance of Tenericutes and Firmicutes was higher in site D fish and site D water samples than other sites. At the genus level, site A fish samples were mostly composed of *Sphingomonas* and *Methylobacterium*. *Sphingomonas* is often isolated from contaminated soils, as members of this genus are able to degrade polycyclic aromatic hydrocarbons (Leys et al., 2004) and are known as microcystin‐degrading bacteria (Hajime et al., 2009; Maruyama, Park, & Ozawa, 2006). *Methylobacterium* is also considered an important emerging genus of microcystin degraders (Kormas & Lymperopoulou, 2013). Interestingly, water samples from site A were also rich in these two genera.

The specific microbial abundance also varied consistently between the water and intestine samples. The mean abundance of *Prevotella* in site A fish was 0.65%, which was significantly higher than that in the other groups. This strain is associated with carbohydrate‐ or fiber‐rich diets in humans (Alauzet, Marchandin, & Lozniewski, 2010; Wu et al., 2011). Since algae were found in the stomach of lake anchovy in summer (Wang, 2016), we assumed that algae at northern Taihu Lake were used as food because of their great number, and the mass propagation of *Prevotella* may help to promote the digestion of algae. We also found high levels of *Microcystis* in the water and intestinal samples from site C. The summer wind speed and direction affect the horizontal distribution of phytoplankton in Taihu Lake, especially *Microcystis* (Huang et al., 2018), which might explain this result. In site D fish, the high abundance of Firmicutes was primarily due to the predominance of *Clostridium* (2.8%), *Lactobacillus* (2.0%), and *Staphylococcus* (0.2%). The eastern region of Taihu Lake is dominated by submerged plants (Zhang et al., 2018) that may increase the pH of the water (Zhao, Zhu, & Mo, 2008), and an increase in soil pH has been shown to strongly stimulate Firmicutes growth (Anderson et al., 2018). *Staphylococcus* abundance usually has a positive correlation with energy intake (Bervoets et al., 2013), which may be due to the abundance of food resources for lake anchovy in the eastern lake region. *Lactobacillus* and *Clostridium* are involved in tryptophan metabolism, the maintenance of the intestinal barrier function, and mucosal homeostasis (Venkatesh et al., 2014; Zelante et al., 2013). The eastern Taihu Lake is moderately nutritive and has not reached the nitrogen concentration range of a cyanobacterial bloom (Lv et al., 2018). Aquatic plants can absorb nitrogen and phosphorus and inhibit algae growth, thus improving the water quality of eastern Taihu Lake, which may cause the increase in probiotics (He, 2008) and *Bacillus* (Qu & Liu, 2002). These plants have different spatial distributions in Taihu Lake. Phytoplankton is the main vegetation type in the northwest region, while submerged vegetation occurs mostly in the eastern region (Qin, 2002, 2009). Previous studies have demonstrated that geographic differences and sampling locations influence the diversity of the fish intestine microbiome (Duan et al., 2017; Roeselers et al., 2011). Thus, it seems that local habitat is a strong driver of variation in the intestinal microbe community of lake anchovy.

### 4.3 Functional analysis

Because the diversity of microbes in the lake anchovy gut may present many important functions that are essential to life, we employed the PICRUSt algorithm to infer potential gene profiles from 16S rRNA sequences. The most abundant functional categories were associated with membrane transport, carbohydrate metabolism, amino acid metabolism, replication and repair, and energy metabolism. The richness of xenobiotic biodegradation and metabolism, energy metabolism, and carbohydrate metabolism richness was greater in larger fish than other fish, reflecting metabolic acceleration as individuals grow (Helland & Grisdalehelland, 1998; Pen‐Hsing & Shi‐Yen, 1993). The abundance of xenobiotic biodegradation and metabolism function in site D fish was significantly lower than that in other sites (*p* < .05). Xenobiotics (chemicals that exist outside the body) may come into contact or even enter the body in some way, and a certain concentration may include toxic effects (Karpouzas & Singh, 2006; Sun, 2010; Wang, Li, & Chang, 2005). Our results indicated that eastern Taihu Lake was a more favorable environment and may provide a suitable habitat for lake anchovy.

In addition, we found that there were some antibiotic‐related bacterial metabolic functions in the intestinal tract of lake anchovy in the third and fourth genome annotation levels. For example, the biosynthesis of vancomycin group antibiotics, beta‐lactam resistance multiple antibiotic resistance protein, and multiple antibiotic resistance protein MarB were found in the gut. However, the abundance of multidrug resistance protein was significantly lower at site D than at the other three sites (ANOVA, *p* < .001), and the microbial metabolism of the four water environments showed the same trend. This phenomenon indicates that there is relatively few bacterial flora that can produce drug‐resistant substances in both lake anchovy and water of the eastern lake area.

Further, ATP‐binding protein of the antibiotic transporting system was significantly higher in fish guts from site D than other sites (ANOVA, *p* ≤ .001), indicating that the defensive ability of the lake anchovy in eastern Taihu Lake was stronger than that of fish from other parts of the lake (Zhao et al., 2018). These data provide further evidence that the eastern Taihu Lake area provides a relatively good environment and abundant food resources for lake anchovy. The discovery of potential antibiotic resistance in this anchovy species will benefit the development and application of antibiotics in practical aquaculture.

However, we should be cautious in our interpretation of the predictive results due to the inherent limitations of PICRUSt (David et al., 2013). Although some functions could be inferred using these predictive methods, many of the actual functions of the gut microbiota still remain to be discovered, with the help of multiple “omics” approaches (Ferrer, Martins Dos Santos, Ott, & Moya, 2013; Waite & Taylor, 2014).

## 5 CONCLUSION

The present study documented the basic composition of the gut microbiota in lake anchovy in Taihu Lake, China, and investigated the potential factors that influence variation in microbial community composition. Our study demonstrated that fish sex, body size, and local habitat had significant effects on the intestinal flora community structure. Sex affected the composition of the intestinal microbial community at lower taxonomic levels, and the diversity of the gut microbiota was lower in larger fish than in other fish. The structure and richness of intestinal microorganisms were also significantly different in different geographic locations, suggesting that diet and water quality also play important roles in determining the intestinal microbial communities of fish. These findings will not only help researchers understand the community composition of the intestinal microflora of lake anchovy but also contribute to the protection of fish resources in Lake Taihu and the sustainable use and harvest of lake anchovy.

## CONFLICT OF INTEREST

The authors declare that they have no conflict of interests.

## AUTHOR CONTRIBUTIONS

MJ, MYX, YPY, PD, and KL conceptualized the study; MJ, YPY, PD, and KY investigated the study; MJ, CPY, and MYX involved in formal analysis; KL involved in funding acquisition; MJ prepared original draft; and MJ, CPY, DHY, and KL wrote review and edited the manuscript.

## ETHICAL APPROVAL

Our sampling was conducted with legal nets during the permitted fishing period. The entire experimental procedure was approved and monitored by the Freshwater Fisheries Research Center, Chinese Academy of Fishery Sciences (FFRC, CAFS).

## Data Availability

All sequences generated by high‐throughput sequencing were submitted to NCBI Sequence Read Archive under the accession numbers SRR8589057‐SRR8589240, SRR8589245‐SRR8589248.

## APPENDIX 1

### 1.1

**Table A1:** Biometric data of all lake anchovy samples

Number	Total length (mm)	Body length (mm)	Body weight (g)	Sex	Number	Total length (mm)	Body length (mm)	Body weight (g)	Sex
AFS‐1	158.10	145.80	8.21	♀	CFS‐1	184.00	163.93	13.08	♀
AFS‐2	163.12	147.16	9.90	♀	CFS‐2	158.94	141.65	8.14	♀
AFS‐3	161.19	145.30	10.20	♀	CFS‐3	177.19	157.17	12.70	♀
AFS‐4	164.22	149.13	12.10	♀	CFS‐4	163.87	145.88	9.48	♀
AFS‐5	181.82	161.91	13.00	♀	CFS‐5	176.21	157.57	11.93	♀
AFS‐6	164.32	148.21	10.52	♀	CFS‐6	186.76	164.95	14.37	♀
AFS‐7	163.32	145.93	10.73	♀	CFS‐7	158.94	143.80	7.35	♀
AFS‐8	170.17	151.08	13.00	♀	CFP‐1	225.03	203.61	30.09	♀
AFP‐1	218.60	201.18	29.81	♀	CFP‐2	218.82	200.27	29.14	♀
AFP‐2	230.00	209.85	30.12	♀	CFP‐3	223.81	201.53	29.98	♀
AFP‐3	238.62	217.11	30.89	♀	CFP‐4	227.66	208.85	31.32	♀
AFP‐4	222.84	203.64	29.30	♀	CFP‐5	228.26	205.01	30.70	♀
AFP‐5	229.27	206.85	29.70	♀	CFP‐6	221.55	199.80	29.38	♀
AFP‐6	232.94	212.20	31.00	♀	CFP‐7	230.02	208.79	30.40	♀
AFP‐7	224.66	205.77	31.00	♀	CFP‐8	222.48	201.71	30.32	♀
AFP‐8	232.82	212.84	31.80	♀	CFL‐1	269.87	239.21	57.51	♀
AFL‐1	261.21	241.23	49.00	♀	CFL‐2	264.98	241.22	53.95	♀
AFL‐2	251.10	230.22	49.46	♀	CFL‐3	252.68	229.57	48.73	♀
AFL‐3	260.97	239.09	53.23	♀	CFL‐4	253.34	228.97	48.39	♀
AFL‐4	263.73	236.41	48.82	♀	CFL‐5	267.00	243.10	55.05	♀
AFL‐5	257.44	237.91	50.16	♀	CFL‐6	258.89	235.38	49.80	♀
AFL‐6	261.77	239.95	50.79	♀	CFL‐7	259.58	236.68	49.80	♀
AFL‐7	262.33	238.03	51.11	♀	CFL‐8	257.84	235.54	48.30	♀
AFL‐8	258.09	233.64	51.48	♀	CMS‐1	161.27	148.61	11.35	♂
AMS‐1	160.57	144.83	9.00	♂	CMS‐2	160.06	143.45	12.89	♂
AMS‐2	170.63	149.49	9.02	♂	CMS‐3	167.24	147.50	11.95	♂
AMS‐3	157.59	142.52	10.00	♂	CMS‐4	158.69	137.23	11.16	♂
AMS‐4	169.06	152.77	10.90	♂	CMS‐5	152.70	141.52	9.46	♂
AMS‐5	168.56	148.17	12.00	♂	CMS‐6	156.80	139.96	10.76	♂
AMS‐6	159.86	146.02	12.02	♂	CMS‐7	157.13	143.57	11.20	♂
AMS‐7	162.73	147.78	12.80	♂	CMP‐1	226.68	203.64	30.34	♂
AMS‐8	165.70	150.03	9.19	♂	CMP‐2	224.33	201.59	30.98	♂
AMP‐1	222.88	203.61	28.30	♂	CMP‐3	223.64	204.67	32.17	♂
AMP‐2	219.06	200.59	26.60	♂	CMP‐4	221.58	198.54	32.17	♂
AMP‐3	222.34	200.10	28.42	♂	CMP‐5	223.45	201.27	27.15	♂
AMP‐4	217.33	197.40	28.70	♂	CMP‐6	227.68	205.76	29.26	♂
AMP‐5	227.26	212.62	30.50	♂	CMP‐7	242.78	222.35	35.80	♂
AMP‐6	238.11	217.60	31.40	♂	CMP‐8	238.90	216.12	36.60	♂
AMP‐7	224.52	202.70	28.86	♂	CML‐1	272.08	244.46	47.68	♂
AMP‐8	242.27	219.25	38.24	♂	CML‐2	275.46	259.11	50.82	♂
AML‐1	255.52	232.74	49.29	♂	CML‐3	269.52	243.11	58.02	♂
AML‐2	261.66	234.62	51.19	♂	CML‐4	265.66	242.75	51.80	♂
AML‐3	269.34	245.43	56.00	♂	CML‐5	264.37	236.27	54.90	♂
AML‐4	261.27	235.99	47.65	♂	CML‐6	281.93	255.15	59.25	♂
AML‐5	260.34	234.01	54.32	♂	CML‐7	258.45	232.31	47.00	♂
AML‐6	264.87	239.30	53.97	♂	DFS‐1	160.89	143.18	9.54	♀
AML‐7	261.52	241.22	55.04	♂	DFS‐2	162.40	146.63	11.66	♀
AML‐8	257.84	224.99	53.00	♂	DFS‐3	159.65	138.91	10.29	♀
BFS‐1	162.96	146.83	9.20	♀	DFS‐4	171.90	151.66	10.97	♀
BFS‐2	163.51	148.79	9.20	♀	DFS‐5	170.32	151.76	10.81	♀
BFS‐3	173.35	155.77	10.2	♀	DFS‐6	163.92	143.90	9.83	♀
BFS‐4	171.16	153.49	10.24	♀	DFS‐7	160.62	144.19	9.86	♀
BFS‐5	169.08	153.15	10.4	♀	DFS‐8	165.15	149.85	10.85	♀
BFS‐6	169.30	152.15	10.97	♀	DFP‐1	223.01	202.29	30.94	♀
BFS‐7	171.63	156.86	11.15	♀	DFP‐2	221.17	202.93	29.65	♀
BFS‐8	161.01	144.89	8.90	♀	DFP‐3	221.83	200.28	31.92	♀
BFP‐1	229.74	205.50	29.22	♀	DFP‐4	214.96	201.66	32.50	♀
BFP‐2	217.85	198.12	29.25	♀	DFP‐5	222.98	204.08	31.20	♀
BFP‐3	217.17	199.84	29.38	♀	DFP‐6	206.38	188.40	33.00	♀
BFP‐4	225.90	205.84	29.55	♀	DFP‐7	232.03	211.80	31.98	♀
BFP‐5	224.16	203.64	30.14	♀	DFP‐8	222.13	207.95	30.56	♀
BFP‐6	227.38	207.32	30.80	♀	DFL‐1	272.82	246.76	55.60	♀
BFP‐7	230.64	209.55	32.53	♀	DFL‐2	269.20	245.71	61.16	♀
BFP‐8	229.99	208.76	33.60	♀	DFL‐3	271.66	255.62	61.30	♀
BFL‐1	271.70	245.92	58.96	♀	DFL‐4	249.65	225.47	51.20	♀
BFL‐2	258.39	235.43	50.02	♀	DFL‐5	271.43	245.97	61.86	♀
BFL‐3	269.52	243.32	60.25	♀	DFL‐6	247.42	227.01	49.60	♀
BFL‐4	275.03	253.66	55.21	♀	DFL‐7	254.51	231.10	52.33	♀
BFL‐5	264.83	243.37	51.20	♀	DFL‐8	257.80	241.61	63.50	♀
BFL‐6	268.77	242.99	51.84	♀	DMS‐1	169.77	157.00	11.48	♂
BFL‐7	264.80	240.63	54.40	♀	DMS‐2	173.02	152.65	11.34	♂
BFL‐8	275.45	250.78	52.80	♀	DMS‐3	169.67	159.26	14.11	♂
BMS‐1	168.62	154.92	8.67	♂	DMS‐4	164.71	147.26	11.92	♂
BMS‐2	170.96	154.16	8.76	♂	DMS‐5	170.12	152.31	14.40	♂
BMS‐3	171.39	155.24	9.77	♂	DMS‐6	151.56	134.77	9.00	♂
BMS‐4	176.34	158.53	10.80	♂	DMS‐7	171.68	154.31	13.54	♂
BMS‐5	176.17	159.77	11.20	♂	DMP‐1	221.13	207.72	31.99	♂
BMS‐6	177.07	157.70	11.26	♂	DMP‐2	229.48	207.92	33.00	♂
BMS‐7	178.89	161.81	11.60	♂	DMP‐3	229.61	205.22	31.71	♂
BMP‐1	238.76	208.62	28.20	♂	DMP‐4	227.98	206.71	33.10	♂
BMP‐2	243.66	214.62	28.50	♂	DMP‐5	217.38	197.74	33.15	♂
BMP‐3	244.44	214.72	29.46	♂	DMP‐6	218.24	197.87	32.10	♂
BMP‐4	216.66	199.03	25.12	♂	DMP‐7	207.50	188.83	27.80	♂
BMP‐5	218.43	199.10	25.24	♂	DMP‐8	231.51	206.77	33.40	♂
BMP‐6	223.29	198.57	26.30	♂	DML‐1	244.72	220.98	48.27	♂
BMP‐7	223.86	205.18	27.60	♂	DML‐2	250.97	234.46	53.40	♂
BML‐1	272.95	242.95	50.27	♂	DML‐3	245.35	227.60	52.10	♂
BML‐2	261.94	237.79	49.25	♂	DML‐4	248.26	222.76	52.80	♂
BML‐3	254.81	232.09	45.25	♂	DML‐5	269.59	246.17	50.27	♂
BML‐4	253.99	231.47	45.67	♂	DML‐6	270.00	244.31	54.80	♂
BML‐5	257.61	233.49	48.38	♂	DML‐7	239.65	216.11	46.04	♂
BML‐6	277.29	247.45	51.39	♂	DML‐8	244.00	220.68	48.79	♂

Note

Three‐letter codes for each column indicate sample point, fish sex and fish body size. Fish were grouped by sex (F = female, M = male); body size (S = small, about 10 g, P = medium, about 30 g, and L = large, about 50 g); and lake region (A = northern part of Taihu Lake, B = western, C = southern, D = eastern). For example, AFS = fish from site A, of female sex, and small size. The water column (farthest right) shows samples from each site A, B, C, and D.

**Table A2:** The number of effective sequences and OTU Statistics of each sample

Sample name	Effective sequence number	OTU number	Sample name	Effective sequence number	OTU number	Sample name	Effective sequence number	OTU number
AFS‐1	28,960	1,177	BFP‐1	31,640	1,352	CFL‐5	33,336	1,658
AFS‐2	29,603	1,111	BFP‐2	32,210	1,690	CFL‐6	35,296	1,753
AFS‐3	32,521	1,319	BFP‐3	29,141	1,486	CFL‐7	37,847	1,349
AFS‐4	31,646	1,136	BFP‐4	29,405	1,423	CFL‐8	32,350	1,518
AFS‐5	33,612	1,197	BFP‐5	29,975	1,445	CML‐1	28,346	1,446
AFS‐6	32,384	1,196	BFP‐6	35,343	1,572	CML‐2	33,732	1,747
AFS‐7	32,368	1,086	BFP‐7	28,165	1,199	CML‐3	35,232	1,494
AFS‐8	35,175	1,184	BFP‐8	35,571	1,790	CML‐4	33,677	1,358
AMS‐1	28,005	1,368	BMP‐1	33,162	1,458	CML‐5	35,801	1,769
AMS‐2	28,207	1,227	BMP‐2	34,651	1,698	CML‐6	37,805	1,529
AMS‐3	27,616	1,029	BMP‐3	30,125	1,574	CML‐7	38,430	1,691
AMS‐4	27,634	1,065	BMP‐4	32,973	1,576	DFS‐1	46,743	1,858
AMS‐5	29,790	1,059	BMP‐5	32,796	1,532	DFS‐2	46,826	1,934
AMS‐6	27,906	1,104	BMP‐6	32,550	1,688	DFS‐3	43,930	1,661
AMS‐7	27,805	1,021	BMP‐7	30,418	1,706	DFS‐4	39,945	1,589
AMS‐8	31,049	1,095	BFL‐1	36,768	1,291	DFS‐5	39,980	1,428
AFP‐1	42,539	1,373	BFL‐2	27,859	1,491	DFS‐6	39,629	1,671
AFP‐2	40,619	1,239	BFL‐3	31,933	1,392	DFS‐7	37,356	1,685
AFP‐3	39,698	1,544	BFL‐4	31,549	1,384	DFS‐8	36,745	1,760
AFP‐4	38,455	1,810	BFL‐5	29,194	1,603	DMS‐1	39,147	1,597
AFP‐5	38,755	1,687	BFL‐6	28,404	1,461	DMS‐2	31,106	1,082
AFP‐6	39,020	1,892	BFL‐7	28,699	1,520	DMS‐3	39,541	1,465
AFP‐7	68,351	2,170	BFL‐8	33,962	1,238	DMS‐4	29,843	1,252
AFP‐8	38,200	1,653	BML‐1	31,840	1,524	DMS‐5	38,673	1,396
AMP‐1	37,309	1,530	BML‐2	34,002	1,274	DMS‐6	30,376	992
AMP‐2	36,849	1,635	BML‐3	30,557	1,557	DMS‐7	38,330	1,256
AMP‐3	73,960	2,144	BML‐4	36,194	1,709	DFP‐1	39,569	1,686
AMP‐4	37,549	1,646	BML‐5	29,117	1,529	DFP‐2	43,938	1,694
AMP‐5	37,173	1,607	BML‐6	28,739	1,448	DFP‐3	42,681	1,717
AMP‐6	39,573	1,893	CFS‐1	31,851	845	DFP‐4	37,571	764
AMP‐7	35,637	1,901	CFS‐2	34,121	1,258	DFP‐5	35,588	1,596
AMP‐8	55,840	1,991	CFS‐3	33,524	1,147	DFP‐6	32,132	1,242
AFL‐1	39,304	1,692	CFS‐4	34,720	1,387	DFP‐7	32,560	1,479
AFL‐2	67,093	1,883	CFS‐5	35,643	1,125	DFP‐8	31,025	1,617
AFL‐3	38,577	1,835	CFS‐6	36,050	1,334	DMP‐1	33,782	1,273
AFL‐4	41,315	1,806	CFS‐7	37,418	1,414	DMP‐2	32,078	1,554
AFL‐5	39,531	1,513	CMS‐1	39,715	1,198	DMP‐3	37,732	1,481
AFL‐6	39,111	1,510	CMS‐2	39,703	1,221	DMP‐4	43,693	1,655
AFL‐7	39,448	1,963	CMS‐3	38,167	1,066	DMP‐5	32,997	1,118
AFL‐8	37,687	1,718	CMS‐4	44,509	1,163	DMP‐6	39,887	1,164
AML‐1	67,576	2,018	CMS‐5	32,640	1,086	DMP‐7	36,768	1,479
AML‐2	63,966	2,064	CMS‐6	37,188	1,147	DMP‐8	35,020	890
AML‐3	37,960	1,482	CMS‐7	32,625	1,098	DFL‐1	28,811	1,279
AML‐4	35,611	1,552	CFP‐1	36,595	1,625	DFL‐2	38,366	1,167
AML‐5	38,400	1,475	CFP‐2	30,296	1,605	DFL‐3	34,445	729
AML‐6	28,695	1,452	CFP‐3	36,574	1,432	DFL‐4	34,855	1,023
AML‐7	30,167	1,175	CFP‐4	35,965	1,631	DFL‐5	30,154	1,129
AML‐8	58,061	1,791	CFP‐5	36,205	1,693	DFL‐6	34,254	913
BFS‐1	32,382	1,481	CFP‐6	39,695	1,813	DFL‐7	36,448	710
BFS‐2	31,796	1,327	CFP‐7	36,615	1,666	DFL‐8	32,621	885
BFS‐3	34,913	1,396	CFP‐8	41,279	1,201	DML‐1	42,825	1,334
BFS‐4	33,550	1,385	CMP‐1	36,442	1,262	DML‐2	35,408	1,085
BFS‐5	35,946	1,644	CMP‐2	37,114	1,712	DML‐3	37,690	646
BFS‐6	34,255	1,366	CMP‐3	36,264	1,705	DML‐4	35,405	1,594
BFS‐7	36,399	1,522	CMP‐4	37,713	1,309	DML‐5	36,034	762
BFS‐8	39,017	1,330	CMP‐5	39,190	1,822	DML‐6	32,776	1,656
BMS‐1	34,847	1,291	CMP‐6	35,633	1,623	DML‐7	32,924	1,629
BMS‐2	35,566	1,451	CMP‐7	37,362	1,785	DML‐8	35,645	1,248
BMS‐3	34,784	1,438	CMP‐8	37,338	1,633	BW	47,908	1,138
BMS‐4	29,449	1,179	CFL‐1	30,786	1,458	CW	42,025	986
BMS‐5	27,677	1,193	CFL‐2	37,888	1,741	DW	42,105	1,176
BMS‐6	27,337	1,223	CFL‐3	37,328	1,737	AW	46,036	1,070
BMS‐7	42,740	1,324	CFL‐4	40,860	1,869			

Notes

Three‐letter codes for each column indicate sample point, fish sex and fish body size. Fish were grouped by sex (F = female, M = male); body size (S = small, about 10 g, P = medium, about 30 g, and L = large, about 50 g); and lake region (A = northern part of Taihu Lake, B = western, C = southern, D = eastern). For example, AFS = fish from site A, of female sex, and small size. The water column (farthest right) shows samples from each site A, B, C, and D.
